# P-1667. Effect of Penicillin Allergy on Common Urinary Tract Pathogens within a Community Health-System Antibiogram

**DOI:** 10.1093/ofid/ofae631.1833

**Published:** 2025-01-29

**Authors:** Katherine Weller, Christopher M Bland, Logan C Bradley, Susan E Smith, Bruce M Jones

**Affiliations:** University of Georgia College of Pharmacy, Savannah, Georgia; University of Georgia College of Pharmacy, Savannah, Georgia; St. Jospeh/Candler Hospital, Brooklet, Georgia; University of Georgia College of Pharmacy, Savannah, Georgia; St. Joseph's/Candler Health System, Savannah, GA

## Abstract

**Background:**

A penicillin allergy label promotes use of broad-spectrum and alternative antibiotics, which can cause an increase in resistance. Urinary tract infections are commonly encountered in health systems and few studies have shown how a documented penicillin allergy affects resistance on a yearly antibiogram for these isolates.

Urinary Antibiogram for Penicillin and Non-penicillin Allergic Patients

**Table 1:**
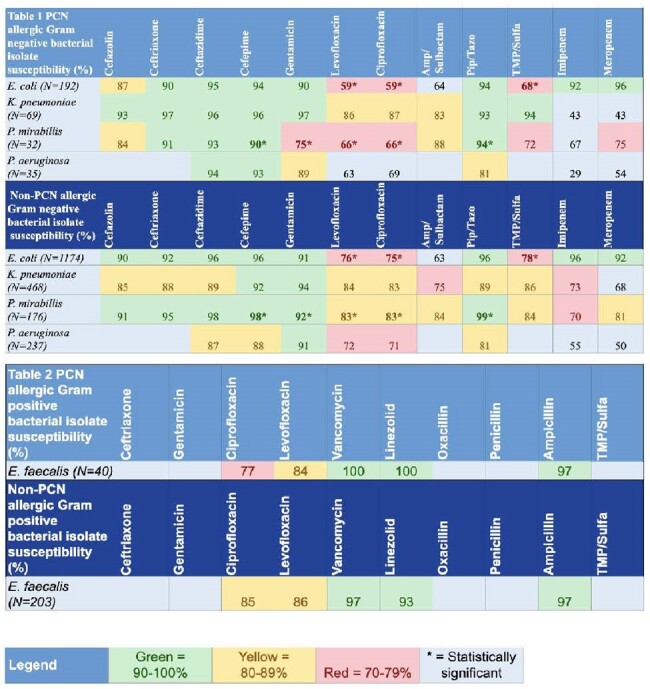
lists Gram negative isolates for penicillin and non-penicillin allergic patients and Table 2 lists the Gram positive isolates

**Methods:**

This was a retrospective, observational chart review of patients admitted in 2022 within a two-hospital community health-system. Using a query language server and third-party software, patients with a first positive bacterial culture were compared with those with a penicillin allergy to create distinct antibiograms. Data collected included culture site and organism, antibiotic susceptibility, and presence of an infectious diseases (ID) consultation. The primary outcome was percentage of susceptible and non-susceptible isolates of *E. coli*, *P. mirabilis*, *K. pneumoniae*, *P. aeruginosa*, and *E. faecalis* in penicillin allergic and non-penicillin allergic patients. A secondary outcome was to determine if ID consultation was different between subgroups. Chi-squared and student's t-test were used for categorical and continuous variables, respectively.

**Results:**

Of 3,757 patients meeting inclusion criteria, 12.8% (n=484) were penicillin allergic with results in Table 1&2. *E. coli* had a significantly lower susceptibility rate for ciprofloxacin (p < 0.001), levofloxacin (p < 0.001), and trim/sulfa (p=0.002) in penicillin allergic patients. *P. mirabilis* had a significantly lower susceptibility rate for cefepime (p=0.015), gentamicin (p=0.006), levofloxacin (p=0.025), ciprofloxacin (p=0.023), and pip/tazo (p=0.012). *K. pneumoniae* showed higher susceptibility rates for multiple antibiotics in the penicillin allergic group. *P. aeruginosa* and *E. faecalis* did not show any statistically significant differences. There was ID consultation on 137/484 (28.3%) of penicillin allergic patients and 899/3273 (27.5%) of non-penicillin allergic patients, which was not statistically different (p=0.7719).

**Conclusion:**

Labeled penicillin allergic patients demonstrated increased resistance in *E. coli* and *P. mirabilis*, while *K. pneumoniae* had higher resistance rates in non-allergic patients. Both groups had similarly high rates of ID consultation.

**Disclosures:**

**Christopher M. Bland, PharmD, FCCP, FIDSA, BCPS**, Merck: Honoraria|Nestle/Seres: Honoraria|Shionogi: Advisor/Consultant|Shionogi: Honoraria **Bruce M. Jones, Pharm.D., FIDSA, BCPS**, AbbVie: Advisor/Consultant|AbbVie: Honoraria|Ferring: Honoraria|Innoviva: Honoraria|Paratek: Honoraria

